# Teflon-buttressed sutures plus pericardium patch repair left ventricular rupture caused by radiofrequency catheter ablation: A case report

**DOI:** 10.1097/MD.0000000000004933

**Published:** 2016-09-23

**Authors:** Hao Cao, Qi Zhang, Yanzhong He, Xiaodong Feng, Zhongmin Liu

**Affiliations:** Department of Cardiovascular Surgery, Shanghai East Hospital, Tongji University School of Medicine, Shanghai, China.

**Keywords:** cardiac tamponade, cardiopulmonary bypass, case report, left ventricular rupture, radiofrequency catheter ablation, surgical treatment

## Abstract

**Background:**

Cardiac rupture often occurs after myocardial infarction or chest trauma with a high mortality rate. However, left ventricular rupture caused by radiofrequency catheter ablation (RFCA) is extremely rare.

**Methods:**

We describe a case of a 61-year-old male who survived from left ventricular rupture caused by a RFCA procedure for frequent ventricular premature contractions. Surgical exploration with cardiopulmonary bypass (CPB) was performed when the signs of cardiac tamponade developed 7 hours after the ablation surgery.

**Results:**

Teflon-buttressed sutures of the tear in the left ventricular posterolateral wall and pericardium patch applied to the contusion region on the wall repaired the rupture safely and effectively.

**Conclusion:**

Timely surgical intervention under CPB facilitated the survival of the patient. Teflon-buttressed sutures plus pericardium patch achieved the successful repair of the rupture.

## 1 Introduction

**Figure 1:**
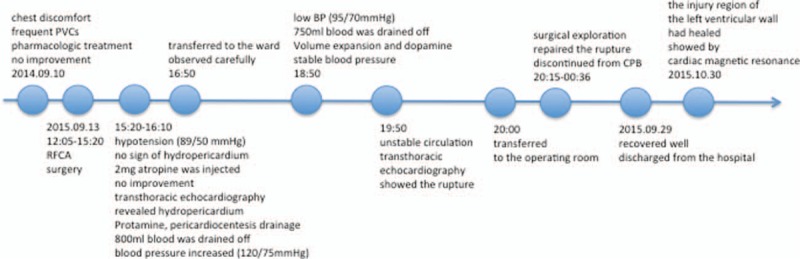
Timeline and information of the case organized into a timeline.

Cardiac rupture is a fatal complication that is associated with high mortality. Left ventricular rupture, usually resulting from myocardial infarction and blunt chest trauma, often leads to sudden death.^[1]^ Cardiac rupture secondary to a radiofrequency catheter ablation (RFCA) procedure is extremely rare, which needs expeditious diagnosis and surgical treatment.^[2]^ We describe a case of a 61-year-old male who survived from left ventricular rupture caused by RFCA. Rapid surgical salvage under cardiopulmonary bypass (CPB) was critically instrumental in saving his life without any complication (Fig. 1).

## 2 Case report

**Figure 2:**
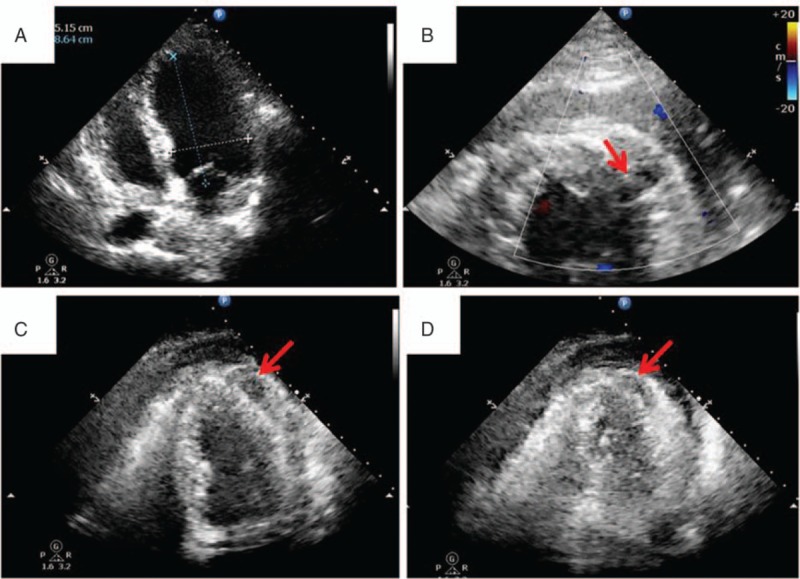
Preoperative cardiac images, (A) transthoracic echocardiography before the radiofrequency catheter ablation (RFCA), (B) transthoracic echocardiography after the RFCA (red arrow: rupture of the left ventricular wall), (C) transthoracic echocardiography after the RFCA (red arrow: hematoma in the left ventricular wall during diastole), (D) transthoracic echocardiography after the RFCA (red arrow: hematoma in the left ventricular wall during systole: smaller than that during diastole due to the contraction of the myocardium).

A 61-year-old male who presented with a 1-year history of recurrent chest discomfort and palpitation was referred to our hospital. Examinations found no positive results except that Holter electrocardiography monitor revealed frequent ventricular premature contractions. He underwent the RFCA procedure since pharmacologic treatment did not show any significant improvement. After RFCA, the patient suddenly experienced dizzy, tight in the chest with cold sweat, bradycardia, and hypotension (89/50 mm Hg). Since chest X ray showed no sign of hydropericardium, vagal reflex was considered first. There was no improvement of blood pressure after rapid fluid infusion, and 2 mg atropine was administered intravenously. Fluoroscopy again showed increased heart shadow, and transthoracic echocardiography revealed hydropericardium. Protamine was administrated intravenously to neutralize heparin. The patient then underwent pericardiocentesis drainage immediately, and 800 mL blood was drained off. Afterward, the chest tightness got better and the blood pressure increased (120/75 mm Hg). Since the patient remained hemodynamically stable for 40 minutes in the catheterization room, he was transferred to the ward and observed carefully. Two hours later, he felt tight in the chest again, blood pressure 95/70 mm Hg, heart rate 85 bpm, and auscultation of the heart found a far low blunt sound. We aspirated the indwelling catheter in the pericardium again and drained off 750 mL blood. Volume expansion and dopamine were administrated to sustain the blood pressure in normal. However, the patient had circulatory unstability again 1 hour later. The transthoracic echocardiography showed a left ventricular wall rupture and a hematoma in the left ventricular wall, which was smaller during systole period due to the contraction of the myocardium (Fig. 2).

**Figure 3:**
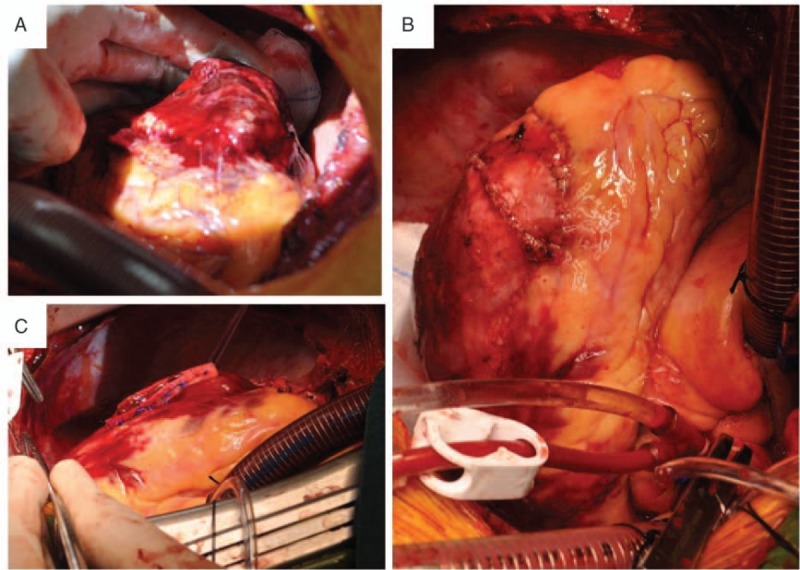
Intraoperative cardiac images, (A) a 6 × 8 cm^2^ area of contusion in the posterolateral wall was found. At the center of the contusion near the apex, a 5-cm tear of the epicardium was revealed, and 1 cm of the tear penetrated into the whole wall. (B) The rupture of the left ventricular wall was repaired by teflon-buttressed sutures. (C) A pericardium patch of sufficient size was applied to overlay the contusion region with interrupted sutures around and bioglue inside.

**Figure 4:**
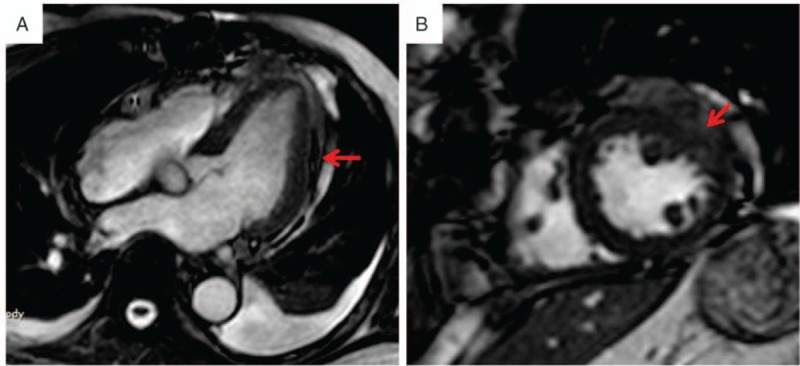
Postoperative cardiac magnetic resonance images, (A) long axis view of cardiac magnetic resonance after surgery (red arrow: repair of the injury region), (B) short axis view of cardiac magnetic resonance after surgery (red arrow: repair of the injury region).

The patient was transferred to the operating room for surgical exploration quickly due to active bleeding of the heart. Median sternotomy was performed. The pericardium cavity was filled with a large amount of blood and clot. After evacuating the pericardium cavity carefully, fresh blood was noted to be coming out of the left side of the heart, and the motion of the left ventricular free wall was hypokinetic. CPB was established with cannulation of the ascending aorta and right atrium quickly. After the cardioplegic arrest, further exploration of the heart revealed a 6 × 8 cm^2^ area of contusion in the posterolateral wall. A 5-cm tear of the epicardium was found at the center of the contusion near the apex, and 1 cm of the tear penetrated into the whole wall. Teflon-buttressed sutures were used to close the tear. Furthermore, a pericardium patch of sufficient size was applied to the contusion region using continuous sutures around and bioglue inside (Fig. 3). The patient was discontinued from CPB uneventfully with excellent hemodynamic status. Postoperatively, he recovered well without any complication and was discharged from the hospital on the 15th postoperative day. A month after discharge, cardiac magnetic resonance showed that the injury region of the left ventricular wall had healed (Fig. 4).

The case study was approved by the Ethic Committee of Shanghai East Hospital, Tongji University School of Medicine, Shanghai, China. Informed consent was obtained from the patient before data collection.

## 3 Discussion

RFCA has emerged as a definitive option to treat patients with cardiac arrhythmia. It is currently being used as a nonpharmacologic alternative for refractory premature ventricular contractions with a beneficial effect.^[3–5]^ RFCA has been reported with a low incidence of complications, among which access site vascular injuries are the most frequent. Other complications including cardiac tamponade, atrioventricular block, thromboembolic event, and coronary artery injuries are quite rare.^[6]^ Cardiac rupture secondary to RFCA is extremely rare that has barely been reported in the literature. Kono et al^[2]^ reported a case of successful salvage of left ventricular rupture caused by RFCA with timely surgical treatment. In their opinion, a small leak from friable myocardium in the ablation site caused oozing into the pericardium cavity and finally progressed to a “blow out” rupture. In our case, the RFCA energy caused damage to myocardium resulting in a contusion region and a slit in the left ventricular wall. Hypotension in the catheterization laboratory was considered for the first time due to hypokinesis of cardiac motion, since no hydropericardium sign was found. Continuous bleeding from the slit resulted in the subsequent hemodynamic instability and further enlargement of the wound.

It is critical to take prompt surgical intervention once conservative treatment did not work. Cautious observation and expeditious diagnosis were needed to recognize the indications for emergency surgical exploration including cardiac tamponade, continuous bleeding, and hemodynamic instability.^[7–10]^ Although sutureless repair without extracorporeal circulation has been reported in cases of cardiac rupture after myocardial infarction recently,^[11,12]^ the classical approach to repair the rupture site under CPB is performed in the life-threatening situation. Since active bleeding of the heart was suspected before the operation, CPB was desperately needed during the surgery for stabilizing the hemodynamic state. Furthermore, an empty, relaxed cardiac condition is much more beneficial for secure repair. After opening the pericardium, fresh blood was noted to be coming out of the heart, and left ventricular rupture was confirmed. During the surgery, CPB guaranteed the safe repair of the rupture and avoided circulatory deterioration. After closing the tear with teflon-buttressed sutures, we applied a pericardium patch to overlay the contusion region to enhance the friable myocardium, which may prevent the ventricular wall from rupturing again.

In conclusion, we describe a case of a 61-year-old male who survived from left ventricular rupture secondary to RFCA. Expeditious surgical intervention under CPB facilitated the survival of the patient without any complication. Teflon-buttressed sutures of the tear and pericardium patch of the contusion region achieved the successful repair of the rupture.

## Acknowledgments

The authors specially thank the staff of the Department of Echocardiography and Radiology for the diagnosis of the disease.
